# Inferences of individual drug responses across diverse cancer types using a novel competing endogenous RNA network

**DOI:** 10.1002/1878-0261.12181

**Published:** 2018-07-14

**Authors:** Yan Zhang, Xin Li, Dianshuang Zhou, Hui Zhi, Peng Wang, Yue Gao, Maoni Guo, Ming Yue, Yanxia Wang, Weitao Shen, Shangwei Ning, Yixue Li, Xia Li

**Affiliations:** ^1^ College of Bioinformatics Science and Technology Harbin Medical University China; ^2^ Bioinformatics Center Key Lab of Systems Biology Shanghai Institutes for Biological Sciences Chinese Academy of Sciences Shanghai China

**Keywords:** ceRNA network, drug response, molecular signature, pan‐cancer analysis

## Abstract

Differences in individual drug responses are an obstacle to progression in cancer treatment, and predicting responses would help to plan treatment. The accumulation of cancer molecular profiling and drug response data provides opportunities and challenges to identify novel molecular signatures and mechanisms of tumor responsiveness to drugs. This study evaluated drug responses with a competing endogenous RNA (ceRNA) system that depended on competition between diverse RNA species. We identified drug response‐related ceRNA (DRCEs) by combining the sequence and expression data of long noncoding RNA (lncRNA), microRNA (miRNA), and messenger RNA (mRNA), and the survival data of cancer patients treated with drugs. We constructed a patient–drug two‐layer integrated network and used a linear weighting method to predict individual drug responses. DRCEs were found to be significantly enriched in known cancer and drug‐associated data resources, involved in biological processes known to mediate drug responses, and correlated to drug activity in cancer cell lines. The dysregulation of DRCE expression influenced drug response‐associated functions and pathways, suggesting DRCEs as potential therapeutic targets affecting drug responses. A further case study in breast invasive carcinoma (BRCA) found that DRCE expression was consistent with the drug response pattern and the aberrant expression of the two NEAT1‐related DRCEs may lead to poor response to tamoxifen therapy for patients with TP53 mutations. In summary, this study provides a framework for ceRNA‐based evaluation of clinical drug responses across multiple cancer types. Understanding the underlying molecular mechanisms of drug responses will allow improved response to chemotherapy and outcomes of cancer treatment.

**Abbreviations**
5‐FU: 5‐Fluorouracil
BLCA: bladder urothelial carcinoma
BRCA: breast invasive carcinoma
ceRNA: competing endogenous RNA
CESC: cervical squamous cell carcinoma and endocervical adenocarcinoma
DRCE: drug response‐related ceRNA
DRS: drug response score
EGFR: Epidermal growth factor receptor
ER: estrogen receptor
GO: gene ontology
HER2: human epidermal growth factor receptor 2
HNSC: head and neck squamous cell carcinoma
IC50: half maximal inhibitory concentration
KEGG: Kyoto Encyclopedia of Genes and Genomes
KIRP: kidney renal papillary cell carcinoma
lncRNA: long noncoding RNA
LUSC: Lung squamous cell carcinoma
miRNA: microRNA
mRNA: messenger RNA
PAS: pathway activity score
PCC: Pearson correlation coefficient
PR: progesterone receptor
RMSE: root ‐mean‐squared error
STAD: stomach adenocarcinoma
TCGA: The Cancer Genome Atlas

## 1 Introduction

The ability to predict the response of individual patients is important for successful cancer treatment. With the development of pharmacogenomics, a large number of cancer molecular profiling and drug response data have been accumulated, which provides opportunities and challenges to identify novel molecular signatures and mechanisms of tumor responsiveness to drugs.

Most previous studies have identified protein‐coding genes as biomarkers of response to drug treatment. Geeleher *et al*. (2014) fitted a ridge regression model of whole‐genome gene expression in cell lines against *in vitro* drug half maximal inhibitory concentration (IC_50_) values and then used the model with tumor expression data from a clinical trial to estimate drug response. Chang *et al*. (2003) identified 92 differentially expressed genes in biopsy samples from primary breast tumor patients before and after docetaxel treatment and used them to predict the response to docetaxel. The importance of noncoding genes, including miRNA and lncRNA, in drug responses has also been demonstrated (Majidinia and Yousefi, 2016; Mishra, 2012). For example, miR‐30c, a prognostic marker in human breast cancer, can control doxorubicin resistance by directly targeting TWF1 and IL‐11, which are two protein‐coding genes that regulate drug sensitivity (Bockhorn *et al*., 2013). The p53‐regulated lincRNA‐p21 plays a physiological role in regulating cell viability following DNA damage (Huarte *et al*., 2010). Nevertheless, these studies did not investigate the interactions between protein‐coding and noncoding RNA.

In a newly proposed ceRNA hypothesis, lncRNA function as decoys that compete for miRNA binding sites and ultimately derepress all target genes of the respective miRNA (Salmena *et al*., 2011). Different species of RNA transcripts can communicate with each other, which have been symbolically referred to as letters of a new language. The competing regulation of lncRNA, miRNA, and gene is not only of fundamental importance in physiological conditions (Cesana *et al*., 2011), but also relevant in various cancers (Wang *et al*., 2015) and may also influence genes associated with drug responses and the development of resistance to cancer therapy (Ling *et al*., 2013). For example, Cao *et al*. (2017) found that the lncRNA, SNHG6‐003, functions as a ceRNA to promote cell proliferation and induce drug resistance in hepatocellular carcinoma, and targeting the ceRNA network involving SNHG6‐003 may be used as a treatment strategy against hepatocellular carcinoma. Zheng *et al*. (2016) demonstrated that ceRNA networks of CYP4Z1 and pseudogene CYP4Z2P confer tamoxifen resistance in breast cancer. Feng *et al*. (2017) indicated that CASC2 up‐regulates PTEN as a ceRNA of miR‐21 and plays an important role in cervical cancer sensitivity to cisplatin. Therefore, understanding this novel RNA cross talk will lead to significant insight into gene interactions and has implication in drug responses.

Here, we performed a systematic analysis to predict clinical drug responses using a ceRNA network consisting of lncRNA, miRNA, and gene competing regulations. DRCEs were identified by combining the sequence, expression, and survival data across various cancer types extracted from The Cancer Genome Atlas (TCGA). Although the DRCEs had a high degree of cancer specificity, they were involved in common biological processes known to mediate drug responses. A patient–drug two‐layer integrated network and a linear weighting method were used to predict drug responses, and the dysregulation of DRCE expression was inferred to trigger functions and pathways associated with differences in the response to drugs. A case study in BRCA was performed to identify DRCEs involved in tamoxifen response. The DRCEs may be potential therapeutic targets to influence individual drug responses. Understanding the underlying molecular mechanisms of drug responses will allow improved response to chemotherapy and outcomes of cancer treatment.

## 2 Materials and methods

### 2.1 Molecular expression and clinical information of cancer patients

Large‐scale mRNA and miRNA expression profiles (Illumina HiSeq level 3), clinical follow‐up survival time, and clinical drug treatment records of cancer patients were obtained from TCGA data portal (Cancer Genome Atlas Research *et al*., 2013). lncRNA expression profiles were retrieved from TANRIC (Li *et al*., 2015), which is an open‐access web resource containing the expression data of lncRNA in large patient cohorts of 20 cancer types, including those in TCGA. Cancer patients with drug treatment that had lncRNA, miRNA, and mRNA expression information, and clinical survival data were retained for subsequent analysis. A total of 13 tumor types include sample sizes of 7–377 patients. For details, see Table S1. To filter lncRNA, miRNA, and genes not expressed across all samples, the items with expression values of 0 in all of the samples were excluded. To allow log transformation, any remaining expression values of 0 were set to the minimum value of all samples and all values were log_2_‐transformed.

### 2.2 Molecular expression and drug activity data of cancer cell lines

The NCI60 is a panel of 60 human cancer cell lines that has been used by the Developmental Therapeutics Program of the U.S. National Cancer Institute to screen over 100,000 chemical compounds for anticancer activity (Shoemaker, 2006). We downloaded the normalized Affymetrix HuEx 1.0 mRNA microarray expression data, Agilent miRNA microarray expression data, and drug activity data measured for IC_50_ for the NCI60 cell lines from cellminer (version 1.6) (Reinhold *et al*., 2012). To assay lncRNA expression, the mRNA expression profile was repurposed by probe level re‐annotation (Du *et al*., 2013). The probe sequences provided by Affymetrix (http://www.affymetrix.com/) were aligned to the lncRNA transcript sequences derived from the Ensembl database (Homo sapiens GRCh37, release 67) (Flicek *et al*., 2012) and Cabili *et al*. (2011) and to protein‐coding and pseudogene transcript sequences derived from the Ensembl (Flicek *et al*., 2012) and UCSC (Kuhn *et al*., 2013) databases. The alignment results were filtered by probes that mapped uniquely and perfectly to lncRNA transcripts, removing probes that mapped to protein‐coding and pseudogene transcripts, and retaining lncRNA covered by at least four probes. A total of 202,449 probes and 10,207 corresponding lncRNA were obtained. If multiple probes corresponded to the same lncRNA, then the average expression value was used to represent the lncRNA expression level.

### 2.3 Method overview

**Figure 1:**
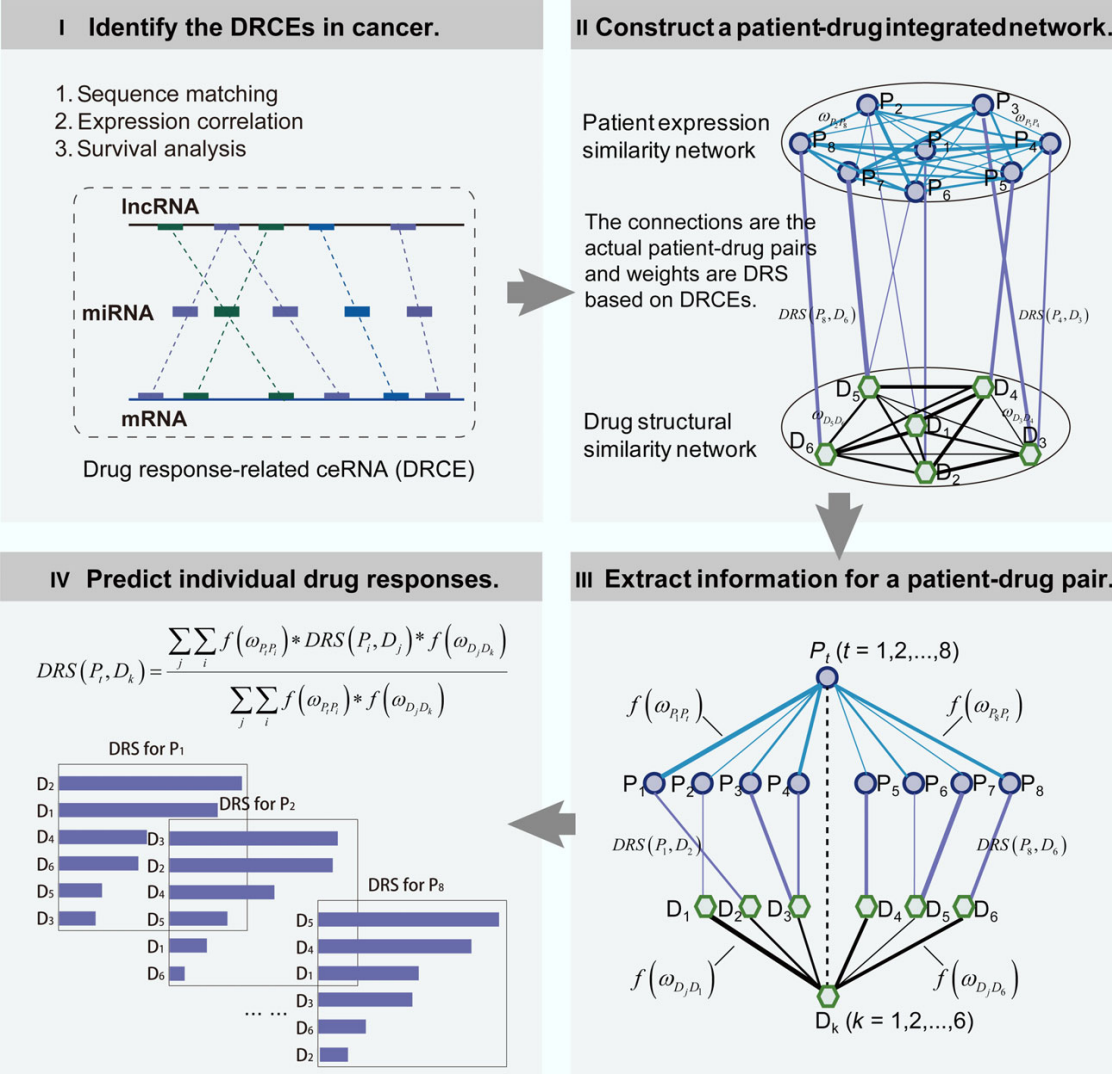
The workflow for predicting individual drug responses based on DRCEs. Step 1: Identification of DRCEs by combining the sequence, expression, and survival data. Step 2: Construction of a patient–drug two‐layer integrated network. Step 3: Extraction of information for scoring the DRS of a particular patient–drug pair. Step 4: Prediction of individual drug responses using a linear weighting method.

The workflow was divided into four phases and is shown in Fig. 1. First, DRCEs were identified by combining the sequence and expression data of lncRNA, miRNA, and mRNA and the survival data of patients treated with drugs. Second, a patient–drug two‐layer integrated network was constructed. The upper layer was a patient expression similarity network, and the lower layer was a drug structural similarity network. The upper and the lower networks were linked using the actual patient–drug relationships retrieved from clinical drug treatment records, and each edge weight was drug response score (DRS) based on the DRCEs. Third, to score a particular patient–drug pair (*P*_*t*_, *D*_*k*_), the patient sample similarity between the query patient and all patients was extracted directly from the patient expression similarity network. The drug similarity between the query drug and all drugs was extracted directly from the drug structural similarity network. Fourth, a linear weighting method was used to predict individual drug responses. The responses of each patient to all drugs were calculated and assigned as the DRS. All drugs were ranked based on the DRS.

### 2.4 Identification of DRCEs across various cancer types

Identifying cancer DRCEs began with screening the ceRNA, which referred to a triplet of a lncRNA, an miRNA, and a gene. We identified ceRNA by reference to previous studies (Ala *et al*., 2013; Karreth *et al*., 2011; Sumazin *et al*., 2011; Tan *et al*., 2015). The miRNA–lncRNA and miRNA–gene interactions were obtained by combining sequence matching and expression negative correlation. The ceRNA triplet consisted of miRNA–lncRNA and miRNA–gene interactions sharing at least one miRNA. The expression of lncRNA and mRNA in a ceRNA triplet was positively correlated with each other. For more details, see in supplementary data.

Response to drug treatment is generally characterized by an increase in patient survival (Lei *et al*., 2013; Pazdur, 2000; Zheng *et al*., 2015). In this study, ceRNA associated with survival of patients treated with drug were considered to be DRCEs. The association of survival time and the expression of lncRNA, miRNA, and genes in ceRNA were tested by univariate Cox regression analysis. The association between survival and each ceRNA was scored by combining the Cox regression *P*‐values of the lncRNA, miRNA, and gene in individual ceRNA using Fisher's combined probability test method (Hwang *et al*., 2005). The score of each ceRNA was calculated as:

$$\text{score}=-2\sum_{i\;=\;1}^{k}\text{log}\left(p_{i}\right)$$

where *k *= 3 represents three nodes in a ceRNA, an lncRNA, an miRNA, and a gene, and *p*_*i*_ is the *P*‐value of node *i* from univariate Cox regression analysis. The score ~ $\chi_{2k}^{2}$ determined the association significance *P*‐value for χ^2^. The DRCEs were defined using a *P*‐value threshold of 0.05.

### 2.5 Proposing a drug response scoring system

We proposed a drug response scoring system based on the DRCEs. The DRS was calculated by considering the cumulative effect of lncRNA, miRNA, and mRNA with competing interactions in the DRCEs. For each cancer type, each nonredundant node in all DRCEs was assigned a Cox regression coefficient. A positive regression coefficient indicated that increased expression was associated with poor survival, and a negative regression coefficient indicated that increased expression was associated with good survival. The DRS for each patient was calculated taking into account both the strength and positive or negative association of lncRNA, miRNA, and genes of all DRCEs with survival.

$$\text{DRS}=-\sum_{i\;=\;1}^{n}\beta_{i}*{\text{Exp}}_{i}$$

where *n* was the number of nonredundant nodes in all DRCEs in the given cancer, β_*i*_ was the univariate Cox regression coefficient of node *i* in the DRCE (Wang *et al*., 2015, 2017), and Exp_*i*_ was the expression level of node *i* of the given patient. Patients were stratified into high or low‐DRS groups using the median as the cutoff. Overall survival in both groups was estimated by the Kaplan–Meier method, and statistical significance was assessed using the log‐rank test. Survival analysis was performed using R package ‘survival’.

### 2.6 Constructing patient–drug two‐layer integrated network

LncRNA, miRNA, and gene expression, drug structure, and clinical drug treatment records were combined to construct the patient–drug two‐layer integrated network that could be used to predict individual drug responses.

We curated the records of drug treatments from TCGA clinical data. In the ‘clinical_drug_cancer.txt’ table, each row or entry recorded one pair of patient and drug. After deleting the pairs with missing drug name, we manually standardized the drug names according to NCI drug dictionary and DrugBank (Wishart *et al*., 2006). To eliminate the influence of multiple drugs on the survival and simplify the drug response prediction model, we chose the records of those patients who corresponded to one drug, established the list of actual patient–drug pairs, and annotated first‐line, second‐line, or later therapy in each cancer (Table S2).

The upper of the two layers was a sample expression similarity network that was a complete graph. For each cancer type, the PCC between any two samples at lncRNA, miRNA, and gene expression levels was calculated, respectively. The edge weight was the minimum PCC to ensure a strict sample correlation. The lower layer was a drug structural similarity network that was also a complete graph. The chemical structures (SDF files) of the drugs were downloaded from the PubChem database (Wang *et al*., 2009). Taking the SDF files as input, drug properties served as numerical molecular descriptors were obtained using the PaDEL‐Descriptor with default settings (Yap, 2011). The edge weight was the PCC of the numerical molecular descriptors of drugs. Using actual patient–drug pairs extracted from treatment records, the upper and the lower layer network were connected. The edge weight was the DRS of a patient to a drug.

### 2.7 Predicting individual drug responses

It was hypothesized that cancer patients with similar expression patterns would respond similarly to drugs having similar chemical structures (Zhang *et al*., 2015). Using the patient–drug two‐layer integrated network, a linear weighting method was developed to predict the DRS of patient *P*_*t*_ to drug *D*_*k*_ based on an actual patient–drug relationship (*P*_*i*_, *D*_*j*_) obtained from clinical treatment records.

$$\text{DRS}\left(P_{t},D_{k}\right)=\frac{\sum_{j}\sum_{i}f\left(\omega_{P_{t}P_{i}}\right)*\text{DRS}\left(P_{i},D_{j}\right)*f(\omega_{D_{j}D_{k}})}{\sum_{j}\sum_{i}f\left(\omega_{P_{t}P_{i}}\right)*f\left(\omega_{D_{j}D_{k}}\right)},$$

where DRS (*P*_*t*_, *D*_*k*_) was the DRS of patient *P*_*t*_ to drug *D*_*k*_ *,* $\omega_{P_{t}P_{i}}$ and $\omega_{D_{J}D_{k}}$ were the edge weights of *P*_*t*_ − *P*_*i*_ and *D*_*j*_ − *D*_*k*_ in the network which were converted by function $f\left(\omega_{P_{t}P_{i}}\right)=e^{-({\left(1-\omega_{P_{t}P_{i}}\right)}^{2}/\left(2\sigma^{2}\right))},f\left(\omega_{D_{j}D_{k}}\right)=e^{-({\left(1-\omega_{D_{j}D_{k}}\right)}^{2}/\left(2\sigma^{2})\right)}$, and σ was a parameter controlling the rate of variation in edge weight.

The leave‐one‐out method was used to determine the parameter σ and to assess the prediction performance. Each actual patient–drug pair was singled out in turn and the remaining pairs used to predict the one that was left out. σ was ranged from 0 to 1, and increment size was 0.001. For example, when σ was 0.001, a linear weighting method was used to predict DRS of each actual patient–drug pair *i*, which was represented by ${\text{predicted}}_{{\mathrm{DRS}}_{i}}$ , and then, compared with the corresponding observed DRS and calculated the root‐mean‐squared error (RMSE), as follows:

$$\text{RMSE}=\sqrt{\frac{\sum_{i=1}^{n}{\left({\text{predicted}}_{{\mathrm{DRS}}_{i}}-{\text{observed}}_{{\mathrm{DRS}}_{i}}\right)}^{2}}{n}},$$

where *n* represented the number of actual patient–drug pairs. With the change of σ, different RMSE values were obtained. The parameter σ was optimized by minimizing RMSE. After determining σ, the prediction performance was evaluated using the PCC of the predicted and observed DRS of all actual patient–drug pairs. A high correlation indicated good prediction performance of this approach.

### 2.8 Measurement of pathway activities in each patient

To investigate whether DRCEs affected the activity of pathways, we used a gene expression metric to identify pathways associated with DRCE. For each cancer, given a gene *i*, let ${\overset{¯}{X}}_{\mathrm{ij}}$ be the expression value for gene *i* in sample *j*. For sample *j*, ${\overset{¯}{X}}_{\mathrm{sj}}$ represented the mean of *X*_*ij*_ over the member genes in pathway *s*, and ${\overset{¯}{X}}_{j}$ represented the mean expression level of all genes detected. The pathway activity score (PAS) of pathway *s* in the sample *j* was assessed by the following function (Levine *et al*., 2006):

$${\text{PAS}}_{\mathrm{sj}}=\frac{{\overset{¯}{X}}_{\mathrm{sj}}-{\overset{¯}{X}}_{j}}{\sigma_{j}}\sqrt{\left|r\right|},$$

where |*r*| was the number of genes in the pathway *s* and σ_*j*_ was the standard deviation of *X*_*ij*_ of all the genes in the sample *j*. A high, positive PAS indicated that genes in the pathway tended to be highly expressed in the sample, and thus indicated high pathway activity. A low, negative PAS indicated low pathway activity.

### 2.9 Functional analysis of DRCEs

We performed gene ontology (GO) functional enrichment analysis of the genes in the DRCEs to investigate the functional roles of DRCEs in cancers. This analysis was performed using the R package ‘GOstats’ (Falcon and Gentleman, 2007) with the human genome as the reference set and the hypergeometric test to calculate the statistical significance.

## 3 Results

### 3.1 Comprehensive characterization of DRCE networks across diverse cancer types

**Figure 2:**
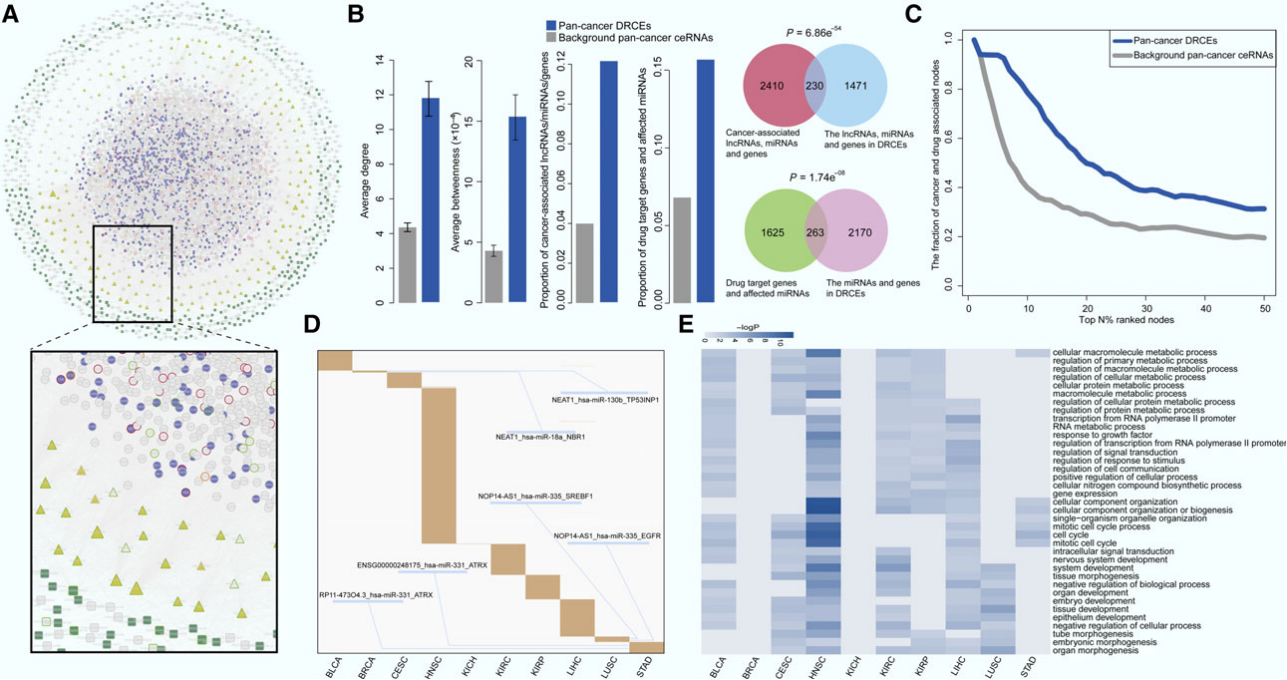
Network and function characteristics of DRCEs. (A) The pan‐cancer ceRNA network is shown as follows: lncRNA as squares, miRNA as triangles, and genes as circles. DRCEs include lncRNA (dark green), miRNA (yellow), and genes (violet). Red borders represent cancer‐associated lncRNA, miRNA, and genes. Light green borders represent drug‐associated lncRNA, miRNA, and genes, and orange borders represent both cancer‐ and drug‐associated lncRNA, miRNA, and genes. Node size represents degree. (B) Multiple topological and functional properties of pan‐cancer DRCEs and ceRNA. (C) The fraction of top nodes of DRCEs (blue) and ceRNA (gray) ranked by the degree that were included in the cancer‐ and drug‐associated lncRNA, miRNA, and gene set. (D) DRCEs (rows) across 10 cancer types (columns). Several DRCEs are indicated as examples. (E) GO functional enrichment analysis of the DRCEs in 10 kinds of cancer (*P *< 0.01). The enriched functions that occurred in at least five cancer types are shown.

A total of 49,207 ceRNA were identified in 13 cancer types, 3854 of them were screened as DRCEs in 10 kinds of cancer (Fig. 2A) and were found to include 303 lncRNA, 135 miRNA, and 1173 genes. The DRCE ranking lists based on shared miRNA number in 10 cancers can be seen in Table S3.

Multiple topological and functional properties of the DRCEs were analyzed against the background of a pan‐cancer ceRNA network (Fig. 2B). Two widely used topological properties, degree and betweenness, were calculated to investigate the important roles of DRCEs. Network nodes with high degree are highly connected and considered as hubs (Barabasi and Oltvai, 2004); nodes with high betweenness control the extent of information flow and are referred to as bottlenecks (Yu *et al*., 2007). We found that nodes in the pan‐cancer DRCEs had significantly higher degree (*t*‐test *P* = 1.10e‐12) and betweenness (*t*‐test *P* = 1.21e‐08) than those in the background pan‐cancer ceRNA. This comparison indicated that nodes in DRCEs tended to be the network hubs and bottlenecks, implying important functions. Cancer‐associated lncRNA, miRNA, and genes from the Lnc2Cancer (Ning *et al*., 2016), miRCancer (Xie *et al*., 2013), and Cancer Gene Census (Futreal *et al*., 2004) databases, which are all manually curated data resources. First, we compared the proportion of cancer‐associated lncRNA, miRNA, and genes in DRCEs and background ceRNA. The result indicated that the proportion of cancer‐associated lncRNA, miRNA, and genes in DRCEs was significantly higher than background ceRNA (Fisher's exact test, *P* < 2.2e‐16). Moreover, we performed enrichment analysis. The result showed that the lncRNA, miRNA, and genes in DRCEs were significantly enriched in cancer‐associated lncRNA, miRNA and gene set (hypergeometric test *P* = 6.86e‐54), but the background ceRNA were not (hypergeometric test *P* ~1). The results indicated that DRCEs were probably cancer‐associated. Finally, when the FDA‐approved drug target genes from the DrugBank database (Law *et al*., 2014) and drug‐affecting miRNA from the SM2miR database (Liu *et al*., 2013) were analyzed, the proportion of drug target genes and affecting miRNA in the DRCEs was significantly larger than that in the background ceRNA (Fisher's exact test *P* = 2.2e‐16). The miRNA and genes in the DRCEs were also significantly enriched in drug‐associated miRNA and gene set (hypergeometric *P* = 1.74e‐08), but background ceRNA were not (hypergeometric *P* ~1). The results suggested that the DRCEs were likely to be druggable.

Furthermore, the lncRNA, miRNA, and genes of DRCEs with high network degree were significantly enriched in known cancer and drug‐related resources (Fig. 2C). The DRCEs also had a high degree of specificity across different cancers. Most of DRCEs occurred only in one specific cancer type; very few were involved in two kinds of cancer (Fig. 2D). Moreover, examination of the degree distribution of the DRCE network in each cancer type revealed a power‐law distribution, showing that the DRCE networks were scale‐free, similar to most biological networks (Fig. S1). Interestingly, functional enrichment analysis of DRCEs showed that the cancer‐specific DRCEs shared some metabolic and cell cycle activities known to mediate drug responses (Fig. 2E) (Housman *et al*., 2014).

### 3.2 DRCE networks contribute to the prognosis of cancer patients treated with drug therapy

**Figure 3:**
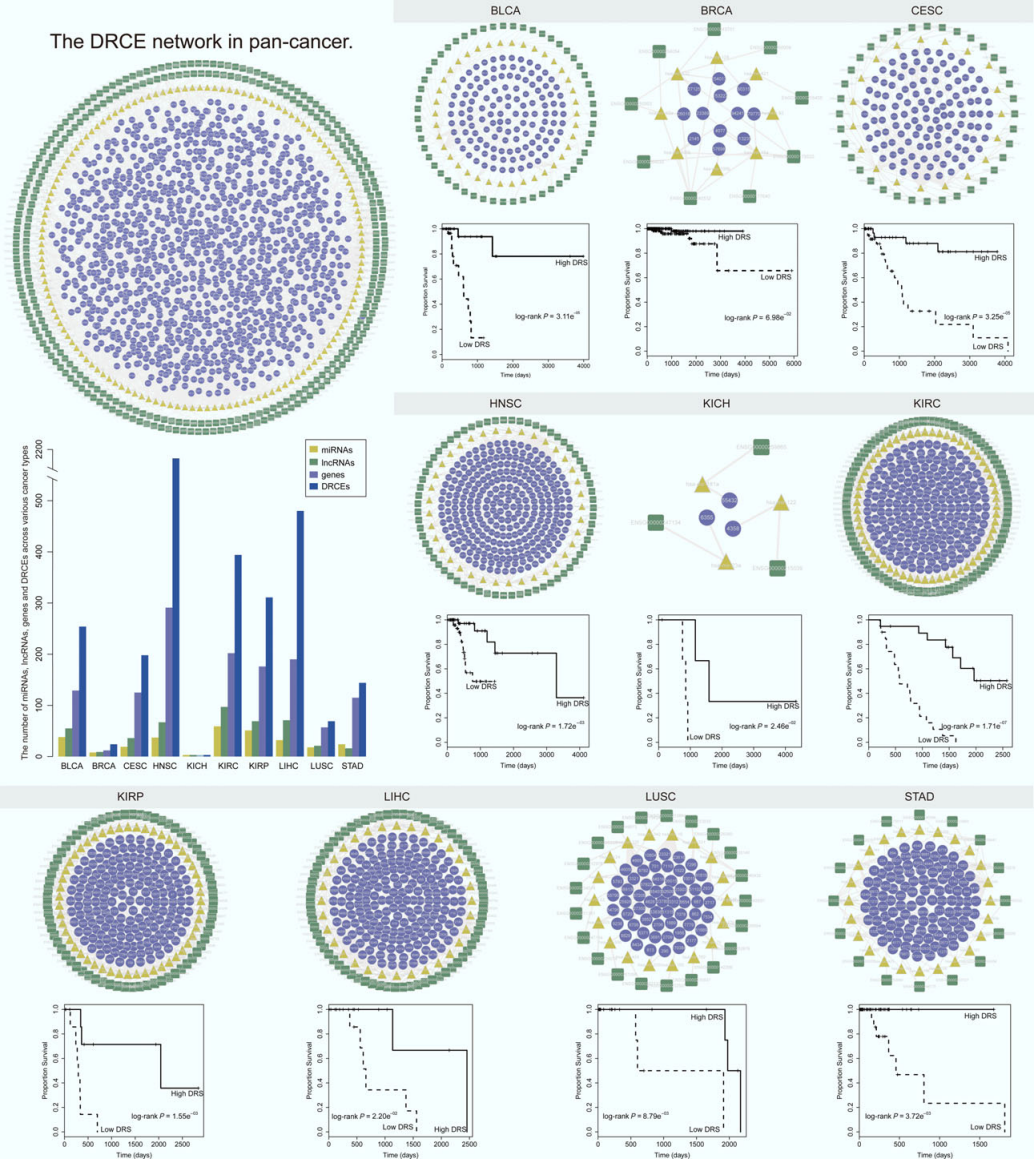
Drug response‐related ceRNA networks as potential drug response signatures had high predictive performance (log‐rank *P *< 0.05). The significance of clinical outcome difference between high‐ and low‐DRS groups was estimated by the Kaplan–Meier method, and the *P*‐value was calculated by the log‐rank test. The DRCE networks in pan‐cancer and individual cancer are shown. Each network is arranged as outer, intermediate, and inner major competing layers. From outside to inside, they are lncRNA (dark green squares), miRNA (yellow triangles), and genes (violet rounds). Edges represent competing regulation. The bar plot shows the number of miRNA, lncRNA, genes, and DRCEs across various cancer types.

Drug responses can be characterized by survival time, which is increased by effective treatment (Lei *et al*., 2013; Pazdur, 2000; Zheng *et al*., 2015). If DRCEs could be used to stratify prognosis, then that would mirror their potential drug response signatures. Notably, the survival analysis showed that the DRCEs successfully characterized patients into different prognosis groups in almost all the cancer types (Fig. 3). Patients with a high DRS tended to have a good prognosis; those with a low‐DRS tended to have poor prognosis. These results indicated that the DRCE networks might act as potential signatures of drug responses.

### 3.3 The DRCEs are correlated to drug activity in cancer cell lines

**Figure 4:**
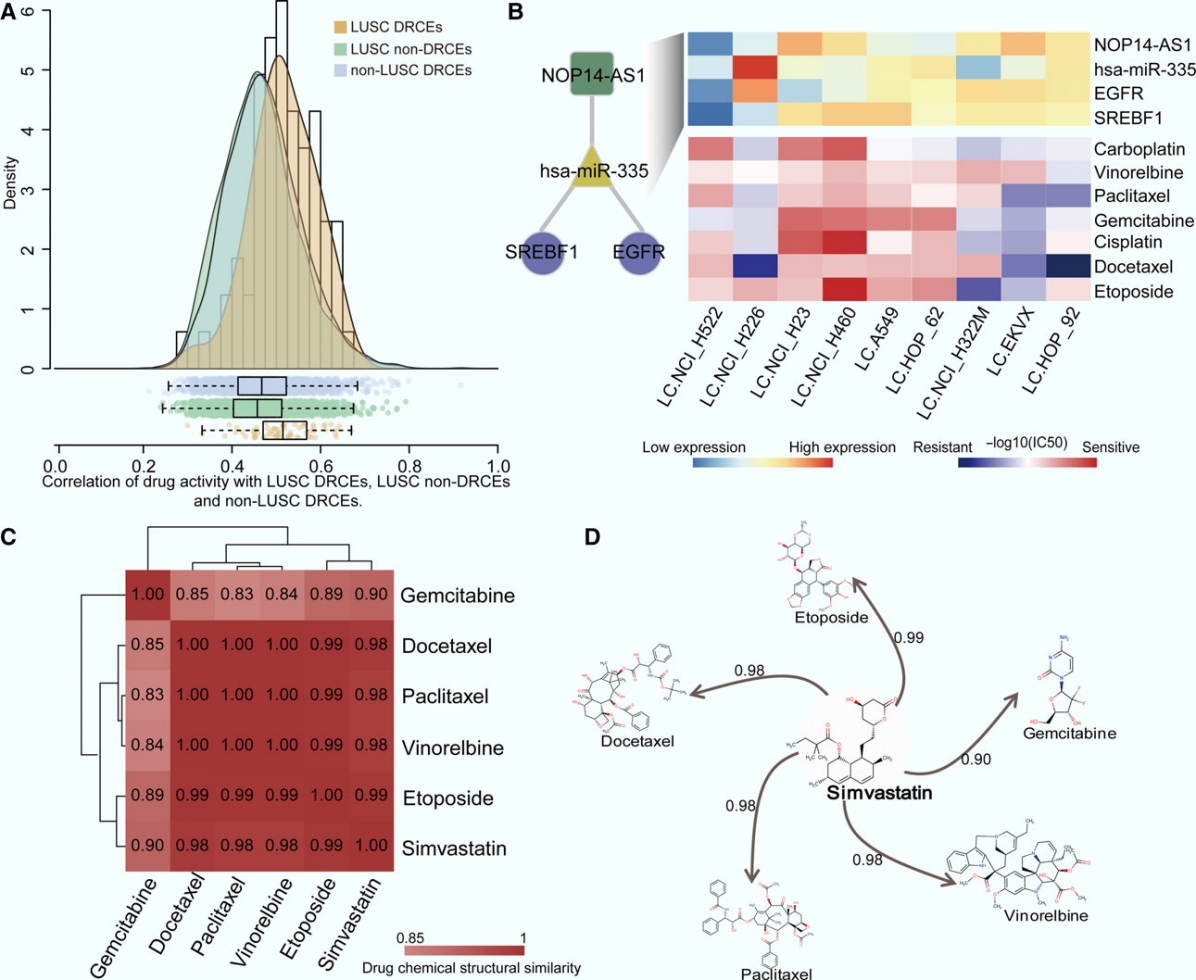
Correlation of DRCEs and drug activity and its use for drug repurposing. (A) Correlation‐density curves and box plots of LUSC DRCEs, LUSC non‐DRCEs, and non‐LUSC DRCEs. (B) Expression of two DRCEs, NOP14‐AS1_hsa‐miR‐335_SREBF1 and NOP14‐AS1_hsa‐miR‐335_EGFR (top), and the activity values (−log_10_ IC_50_) of seven lung cancer drugs (bottom) across nine lung cancer cell lines. Numbers in the matrix (C) and on the line (D) indicate the structural similarity of two drugs.

The NCI60 cancer cell line collection is widely used as a panel to evaluate *in vitro* drug activity (Shoemaker, 2006), with the –logIC_50_ reflecting the drug sensitivity and resistance. The drug activity and expression data of lung cancer cell line could be derived from NCI60. Here, lung cancer cell lines were chosen to assess the correlation of DRCE expression and drug activity, which would further test the relevance of DRCEs to drug responses. We extracted lncRNA, miRNA, mRNA expression, and drug activity values of lung cancer cell lines. A total of 69 DRCEs were identified using TCGA lung squamous cell carcinoma (LUSC) data, 65 of them had expression data in lung cancer cell lines. In TCGA LUSC drug treatment records, seven drugs (carboplatin, vinorelbine, paclitaxel, gemcitabine, cisplatin, docetaxel, and etoposide) had activity data. The mean absolute PCCs of drug activity and lncRNA, miRNA, and gene expression of each DRCE across nine lung cancer cell lines were calculated, and a DRCE was considered to be associated with drug responses if it was correlated with at least one of the seven drugs. Nearly, all the DRCEs (98%) were correlated with at least one drug with a PCC ≥0.3, 94% had PCCs ≥0.4, and 60% had PCCs ≥0.5. In addition, 2335 LUSC non‐DRCEs, which were ceRNA that did not contain nodes in LUSC DRCEs, and 3388 non‐LUSC DRCEs, which were DRCEs in cancers other than LUSC and did not contain nodes in LUSC DRCEs, were also evaluated. LUSC DRCEs had a significantly higher correlation with drug activity than LUSC non‐DRCEs (*t*‐test *P* = 4.40e‐07) and non‐LUSC DRCEs (*t*‐test *P* = 2.10e‐05, Fig. 4A). The results suggest that DRCE expression was associated with *in vitro* drug activity in cancer cell lines. Through literature review, we found two DRCEs NOP14‐AS1_hsa‐miR‐335_EGFR and NOP14‐AS1_hsa‐miR‐335_SREBF1 were associated with lung cancer drug responses (Fig. 4B). Luo *et al*. (2017) reported hsa‐miR‐335 served as a critical regulator of chemo/radio resistance in lung cancer; Huang *et al*. (2012) found that interaction of ΔNp63α and apoptosis‐inducible SREBF1 proteins influenced cisplatin chemoresistance of LUSC. Epidermal growth factor receptor (EGFR) mutations are prevalent and well characterized in lung cancer and were shown by Gazdar (2009) to be associated with sensitivity and resistance to lung cancer treatment. In addition, we performed GO functional annotation analysis using David (Huang da *et al*., 2009) and found that EGFR and SREBF1 were annotated with drug responses related GO terms (Table S4), such as DNA repair, cell proliferation, and cellular response to drug. Thus, we inferred that EGFR and SREBF1 condition drug responses through competing regulation of NOP14‐AS1 mediated by hsa‐miR‐335.

Moreover, we checked the correlation of LUSC DRCE expression and activity of all drugs in the NCI60 panel except for seven known clinical lung cancer drugs which were stored in TCGA drug treatment records of lung cancer (shown in Fig. 4B). The average absolute PCC between activity of each drug and expression of lncRNA, miRNA, and gene of each DRCE in lung cancer cell lines was calculated. The result showed that six drugs (vorinostat, celecoxib, lapatinib, vinorelbine, clofarabine, and simvastatin) with PCCs ≥0.4 with about 50% or more DRCEs, in which the first four drugs have been used to treat lung cancer (Altorki *et al*., 2003; Fossella *et al*., 2000; Ramalingam *et al*., 2010; Ross *et al*., 2010). Simvastatin is a lipid‐lowering drug, and previous study has indicated that lipid metabolic pathways may be valuable as lung cancer therapeutic targets (Yano, 2012). The chemical structure of simvastatin is highly similar to that of five of seven drugs (Fig. 4C,D). To test whether the chemical structural similarity is directly correlated to drug activity, for lung cancer cell line, we calculated the correlation of activity of any two drugs and the similarity of chemical structure of any two drugs in NCI60. The result showed that there was significantly positive correlation between chemical structural similarity and drug activity (PCC = 0.1, *P*‐value = 4.89e‐15). In addition, we compared the correlation of simvastatin's drug activities with the expression of DRCEs and non‐DRCEs and found DRCEs had a significantly higher correlation with simvastatin's drug activities than non‐DRCEs (*t*‐test *P* < 0.001, Fig. S2). The percentage of lncRNA, miRNA, and mRNA in DRCEs whose correlation with simvastatin's drug activities more than 0.5, 0.4, and 0.3 were also greater than non‐DRCEs (Fig. S3). Furthermore, the drug activities of simvastatin were differences in the two clusters classified by the expression of the two favorable DRCEs NOP14‐AS1_hsa‐miR‐335_SREBF1 and NOP14‐AS1_hsa‐miR‐335_EGFR (fold change >2), indicating that the expression of the two DRCEs has effect on the efficacy of simvastatin on lung cancer cell lines. However, the efficacy of simvastatin is no difference between the two clusters classified by the non‐DRCEs. Thus, we infer that simvastatin could be used to treat lung cancer. Our method can not only predict drug response, but also optimize candidate drugs for new indications.

### 3.4 Dysregulation of DRCE expression influences drug response‐associated functions and pathways

The drug responses of individual patients in a specific cancer were predicted with a patient–drug two‐layer integrated network. The leave‐one‐out method was used to estimate parameter σ and evaluate predictive performance. The RMSE range with a change in σ is shown in Fig. S4 for 10 types of cancer. A high correlation was found between the observed and predicted DRS. Eight cancers had PCCs ≥0.4, and five had PCCs >0.8; particularly kidney renal papillary cell carcinoma (KIRP) had a PCC >0.9 (Fig. S5). The results indicated that this method had good, generalizable performance, and effectively predicted drug responses.

**Figure 5:**
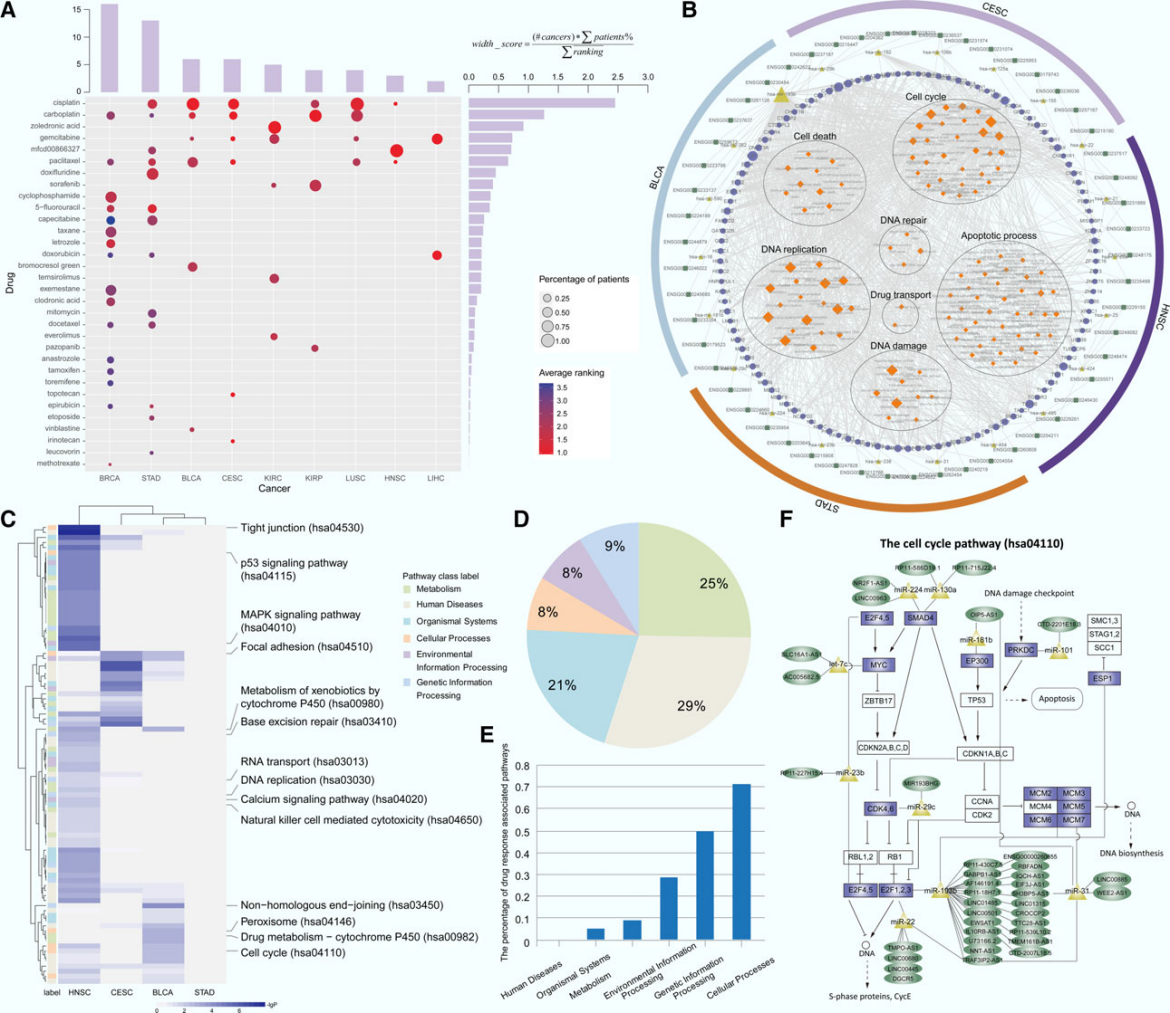
Mechanisms common to drug response differences. (A) The bubble‐bar plot provides an overall view of the predicted high‐DRS drugs ranked at the top 30% (rows) across the nine cancer types (columns). Bubble size represents the percentage of patients at each drug. Bubble color shows the average ranking of each drug for the corresponding patients. The top bars show the number of drugs for a given cancer. Bars on the right show the width score of corresponding drugs. (B) From outside to inside, the functional enrichment results of the dysregulated expression of DRCEs in BLCA, CESC, HNSC and STAD arranged hierarchically with lncRNA, miRNA, genes, and enriched drug response‐associated GO biological process terms. Node size represents degree. (C) Pathway activity difference profile affected by the dysregulated expression DRCEs in BLCA, CESC, HNSC, and STAD. Drug response‐associated pathways are shown. *P*‐value was calculated by *t*‐test to compare the PAS of high‐ and low‐DRS patients treated with cisplatin in four cancers. The label on the left shows six KEGG pathway classes. (D) The percentage of the pathways with differential PAS in the six KEGG pathway classes. (E) The percentage of drug response‐associated pathways in the six KEGG pathway classes. (F) The cell cycle pathway (hsa04110) affected by dysregulated expression of DRCEs in BLCA and HNSC. The rectangles show genes in the pathway, and the genes of DRCEs with dysregulated expression are shown in violet. Dark green ellipses show lncRNA, and yellow triangles show miRNA.

For each patient in each cancer type, drugs were ranked by their predicted DRS in descending order. The drugs with 30% of the highest predicted DRS were taken, acquiring the predicted patient–drug pairs in a given cancer. An overall view of drugs across nine of the cancers is shown in Fig. 5A. To investigate which anticancer drugs could be widely used for cancer treatment, a width score was calculated for each drug taking into account the percentage of patients corresponding to the given drug in each cancer, the average ranking of the given drug for the corresponding patients in each cancer, and the number of cancer corresponding to the given drug. Cisplatin had the highest width score, suggesting that it is preferred for most patients in most cancer types. This study intends to understand the underlying mechanism of individual differences in response to cisplatin. Cisplatin is widely used to treat a variety of cancers and kills cells by directly or indirectly inducing apoptosis, DNA damage, and cell cycle arrest (Siddik, 2003). However, individual differences in response to cisplatin are an obstacle to effective cancer treatment. This study results could be used to understand the underlying mechanism.

The prediction results indicate that cisplatin could be a preferred treatment in six cancer types, and five or more patient–cisplatin pairs were found in four cancers [bladder urothelial carcinoma (BLCA), cervical squamous cell carcinoma and endocervical adenocarcinoma (CESC), head and neck squamous cell carcinoma (HNSC), and STAD]. It is thus important to determine whether any causes of differences in response to cisplatin act across all four cancers. To investigate the functions influenced by cisplatin‐associated DRCEs, first, the patients treated with cisplatin were stratified by DRS with the median as a cutoff value and the differentially expressed lncRNA, miRNA, and genes were determined by *t*‐test (*P* <0.05); then, the cisplatin‐associated DRCEs were screened which contained at least one differentially expressed lncRNA, miRNA, or gene; at last, the function of cisplatin‐associated DRCEs was evaluated by GO functional enrichment analysis (*P*‐value < 0.05). Performing the above analysis for the four cancers, respectively. The results revealed that although the differential genes were different in the four cancers, the functions of the DRCEs are closely associated with drug responses and sensitivity or resistance and have effects on the cell cycle, cell death, apoptosis, DNA damage, replication and repair, and drug transport (Holohan *et al*., 2013; Housman *et al*., 2014). The cisplatin‐associated DRCEs in at least two cancer types are shown in Fig. 5B. The PAS of all Kyoto Encyclopedia of Genes and Genomes (KEGG) pathways in each patient was measured, and those with a PAS that was significantly different in the two patient groups in at least one cancer were retained (Fig. 5C). The 91 pathways in which the human diseases pathway class accounted for the largest proportion are shown in Fig. 5D. A literature search found that the cellular processes pathway class included the most drug response‐associated pathways (Fig. 5E, Table S5). The cell cycle pathway activity included with cellular processes was abnormal in both BLCA and HNSC, and a total of 14 genes of 96 dysregulated DRCEs were in that pathway (Fig. 5F). Therefore, from the results, we could see that the individual differences in drug response to cisplatin might thus be caused by the dysregulation of DRCE expression. Aberrant expression of lncRNA might result in the dysregulation of gene expression by competing for miRNA and ultimately lead to disorders of drug response‐associated functions and pathways. That would make DRCEs potential therapeutic targets to influence response to cisplatin. Understanding novel mechanisms of drug responses allows development of novel treatments that improve the effectiveness of chemotherapy and clinical outcomes of cancer treatment.

### 3.5 Case study: A patient treated with a high‐DRS drug has a good STAD prognosis

**Figure 6:**
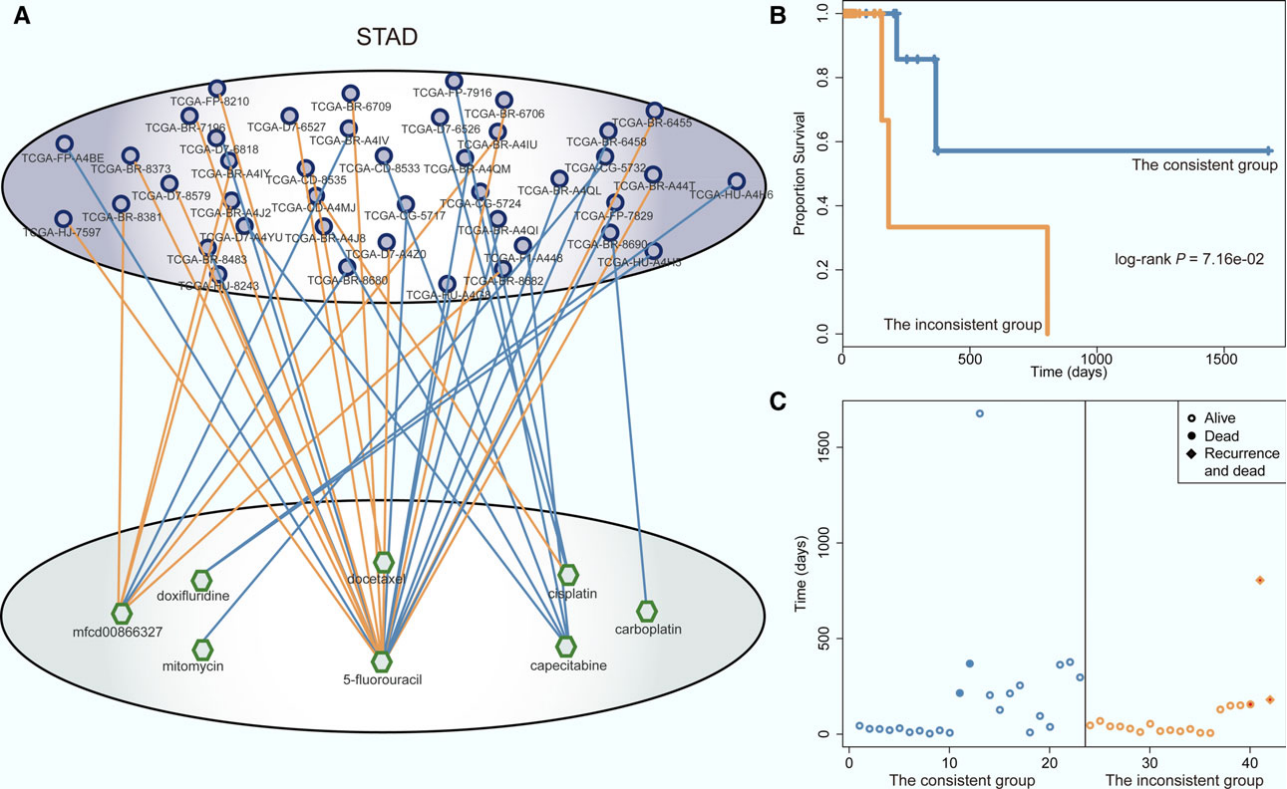
Drug response prediction in STAD. (A) The patient–drug two‐layer integrated network for STAD. The patient–drug pairs of the consistent and inconsistent groups shown in blue and orange edges, respectively. For simplicity, the sample–sample and drug–drug edges are not shown. (B) Kaplan–Meier survival analysis for the consistent (blue) and the inconsistent (orange) groups. The *P*‐value was calculated using the log‐rank test. (C) Survival and status of the consistent (blue) and the inconsistent (orange) groups.

The number of drugs in STAD was the second‐most (Fig. 5A). We further verified whether this approach could offer a promising drug treatment regimen for an individual patient, thereby tailoring the right drug to the right patient in STAD. Based on actual patient–drug pairings in the TCGA clinical drug treatment records, patients who were treated with drugs among those with a DRS within the top 30% were defined as the ‘consistent group’ and those treated with drugs in the bottom 30% were defined as the ‘inconsistent group’. The STAD two‐layer integrated network with the consistent and inconsistent groups and the actual chemotherapy pairings is shown in Fig. 6A. The STAD patients in the consistent group had longer survival and fewer deaths than those in the inconsistent group (Fig. 6B,C), suggesting the effectiveness of a predictive approach using *in vivo* tumors. Fig. 6A shows that most patients were treated with 5‐fluorouracil (5‐FU). ATRX, CCND2, APC, and KLF8 have been implicated in 5‐FU resistance (Chen *et al*., 2009; Conte *et al*., 2012; Schmidt *et al*., 2004; Shi *et al*., 2015). Moreover, the GO functional annotation analysis was performed and found that the four genes were annotated with drug responses related GO terms (Table S6), such as cell cycle, apoptotic process, and DNA damage response. In the STAD DRCEs, four lncRNA, RP11‐473O4.3, LINC01184, LINC00641, and ENSG00000248175, competed for binding to miR‐331, miR‐335, and miR‐106b, which are known to be associated with chemotherapy resistance (Feng *et al*., 2011; Kim *et al*., 2015; Xia *et al*., 2008), thereby regulating their target genes. Consequently, the six DRCEs, hsa‐miR‐335_KLF8_LINC00641, hsa‐miR‐106b_APC_ENSG00000248175, hsa‐miR‐106b_APC_LINC01184, hsa‐miR‐106b_CCND2_ENSG00000248175, hsa‐miR‐331_ATRX_ENSG00000248175, and hsa‐miR‐331_ATRX_RP11‐473O4.3, may affect 5‐FU drug responses (Fig. S6). These findings revealed that the DRCEs could be used clinically to stratify patients to receive specific drug therapeutic targets.

### 3.6 Case study: DRCE expression matches drug response patterns in BRCA

**Figure 7:**
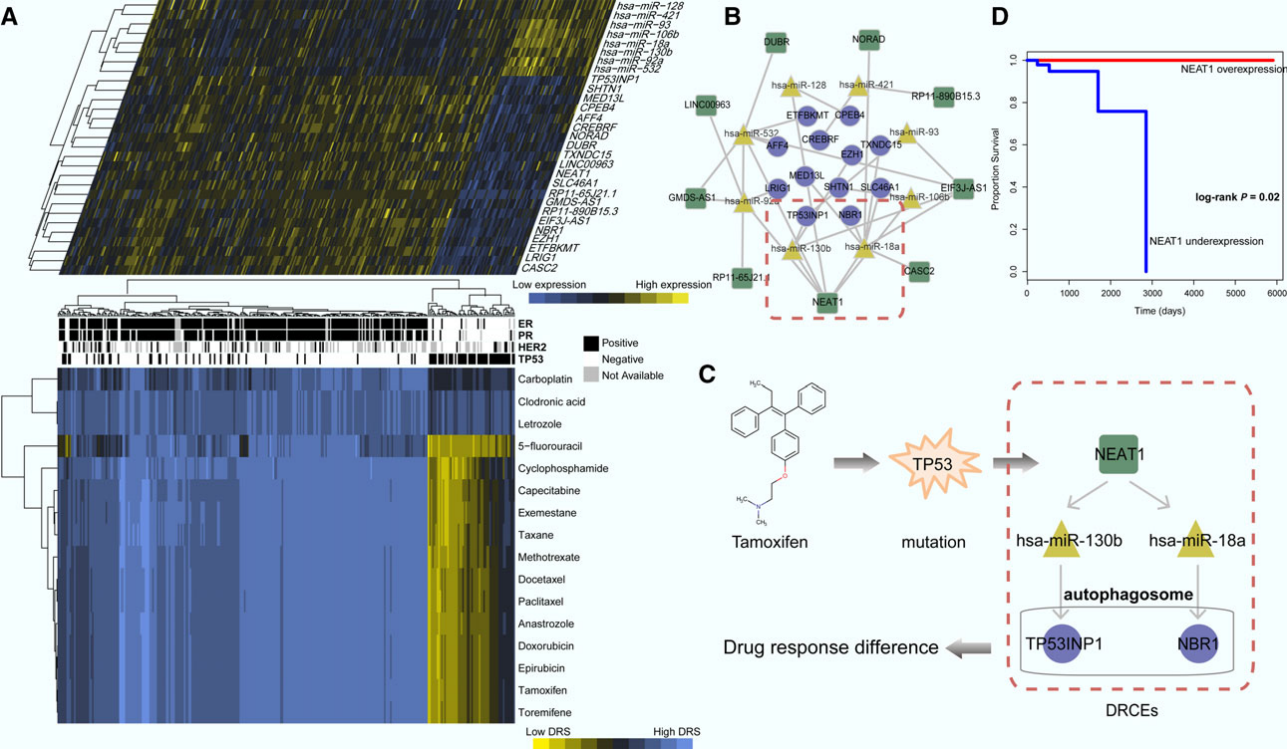
Alignment of DRCE expression and drug response pattern in BRCA. (A) DRCE expression (top panel) and drug response profile (bottom panel). Patients with ER, PR, HER2 subtypes, and TP53 mutation status are shown. (B) The BRCA DRCE network. (C) Two DRCEs, NEAT1_hsa‐miR‐130b_TP53INP1 and NEAT1_hsa‐miR‐18a_NBR1, that influence tamoxifen therapy for BRCA patients with TP53 mutation. (D) The significance of clinical outcome difference between NEAT1 overexpression and underexpression groups was estimated by the Kaplan–Meier method, and the *P*‐value was calculated by the log‐rank test.

In this study, the number of BRCA patients is the most. Among the BRCA patients, 304 were treated with a single drug; 16 drugs were used. The BRCA two‐layer integrated network predicted the DRS of all patients for each drug and hierarchical clustering analysis revealed that the patients and drugs were globally grouped into two classes based on the DRS (Fig. 7A). Differential analysis of lncRNA, miRNA, and gene expression in BRCA DRCEs in the two drug response classes found that all were differentially expressed in the two classes (*t*‐test *P *< 0.05). Currently, treatment decisions are guided by BRCA subtypes that include estrogen receptor (ER), progesterone receptor (PR), and human epidermal growth factor receptor 2 (HER2) status. ER and PR status could be obviously distinguished in the two classes. TP53 mutation is the most frequent genetic alteration in BRCA, and in the 304 patients, ER and PR negative patients with a TP53 mutation had a high DRS to carboplatin, clodronic acid, and letrozole, indicating that the three drugs might be given treatment priority. Tamoxifen is an anti‐estrogen drug that is commonly used to treat ER‐positive patients. ER‐negative patients did not respond well to tamoxifen, which is consistent with the previous studies of breast tumor drug response prediction (Daemen *et al*., 2013). As tamoxifen can target TP53, previous studies found that TP53 mutation can result in tamoxifen resistance in BRCA (Elledge *et al*., 1995). Therefore, we tried to explore the cause of the event based on the involvement of DRCEs.

In cancer therapy, many chemotherapeutic agents cause cell death by damaging DNA in replicating cells. As we known, TP53 is involved in many important cellular responses, such as apoptotic cell death, cell cycle arrest, and DNA repair. A recent study reported that TP53 induced formation of NEAT1 lncRNA‐containing paraspeckles that modulated the replication stress response and chemosensitivity (Adriaens *et al*., 2016). Consistent with this report, TP53 mutation triggered down‐regulation of NEAT1 expression and resulted in poor response to tamoxifen (Fig. 7A). In BRCA, 24 DRCEs were identified involving nine lncRNA, eight miRNA, and 12 genes (Fig. 7B), and NEAT1 were associated with seven DRCEs. Two of them were previously found to be associated with drug responses. They were NEAT1_hsa‐miR‐130b_TP53INP1 and NEAT1_hsa‐miR‐18a_NBR1 (Fig. 7C). Miao *et al*. (2017) reported that hsa‐miR‐130b mediated chemoresistance and proliferation of BRCA cells, and Song *et al*. (2011) reported that hsa‐miR‐18a impaired DNA damage repair. TP53INP1 in autophagosomes was shown to promote autophagy‐dependent cell death (Seillier *et al*., 2012), and NBR1 promotes autophagosomal degradation of ubiquitinated substrates (Kirkin *et al*., 2009). The induction of autophagy in response to therapeutics can be seen as having a prodeath or a prosurvival function that contributes to anticancer efficacy and drug response (Sui *et al*., 2013). The expression dysregulated DRCEs NEAT1_hsa‐miR‐130b_TP53INP1 and NEAT1_hsa‐miR‐18a_NBR1 are thus likely to lead to poor response to tamoxifen therapy for patients carrying TP53 mutations. We further explored how the survival of the 304 BRCA patients with drug treatment changes with the expression of NEAT1. Patients were stratified into two groups using first and third quartile of NEAT1 expression as the cutoff. The survival analysis showed that NEAT1 expression successfully characterized patients into different prognosis groups (log‐rank *P* = 0.02, Fig. 7D), indicating that NEAT1 expression could impact on drug response of BRCA patients.

## 4 Discussion

Chemotherapy is currently the primary treatment for cancer, but its effectiveness is limited by individual differences in drug responses. Therefore, how to evaluate individual drug responses is an urgent need for cancer treatment. Most current studies have predicted drug responses using molecular biomarkers including protein‐coding mRNA or noncoding RNA (such as miRNA and lncRNA). However, the interaction of different RNA species as described by the ceRNA hypothesis has broadened the scope of investigations to include the effects that lncRNA, miRNA, and gene have on drug responses. This study predicted individual drug response based on the ceRNA network across various cancer types.

Our results revealed that cancer and drug‐associated data resources were enriched in the pan‐cancer DRCE network, in which lncRNA, miRNA, and genes also tended to be hubs and bottlenecks. The DRCEs emerged as potential drug response signatures and had high specificity in different cancers but shared many common drug response‐related biological functions. Furthermore, the DRCEs correlated with *in vitro* drug activity in cancer cell lines were applied to drug repurposing or supported new indications. In addition, the performance of the patient–drug two‐layer integrated network was generalizable and could be used to estimate drug responses effectively. Furthermore, we focused on cisplatin which is widely used to treat a variety of cancers and kills cells by directly or indirectly inducing apoptosis, DNA damage, and cell cycle arrest (Siddik, 2003). However, individual differences in response to cisplatin are an obstacle to effective cancer treatment. This study results could be used to understand the underlying mechanism. Our results revealed that differences in individual drug response to cisplatin might be triggered by dysregulation of DRCE expression. Aberrant expression of lncRNA might result in the dysregulation of gene expression by competing for miRNA and ultimately lead to disorders of drug response‐associated functions and pathways. That would make DRCEs as potential therapeutic targets to influence response to cisplatin. Understanding novel mechanisms of drug responses allows development of novel treatments that improve the effectiveness of chemotherapy and clinical outcomes of cancer treatment. We inferred that differences in individual drug responses might be triggered by dysregulation of DRCE expression, ultimately leading to abnormalities of drug response‐associated functions and pathways. The STAD case study patient survival and status results demonstrated the effectiveness of this approach using *in vivo* tumor and treatment characteristics. Two DRCEs, NEAT1_hsa‐miR‐130b_TP53INP1 and NEAT1_hsa‐miR‐18a_NBR1, were found that may modulate the effect of tamoxifen therapy in BRCA patients with TP53 mutation. The lncRNA NEAT1 as a promising target might indirectly regulate TP53INP1 and NBR1 by competing for hsa‐miR‐130b and hsa‐miR‐18a. These findings might be useful for the development of novel drugs that target NEAT1 for use in combination with tamoxifen to prevent or delay resistance.

This study opens a new avenue to leverage publicly available molecular data to evaluate clinical drug responses and contributes to realizing personalized medicine. Compared with conventional chemotherapy, personalized medicine may result in delivery of more effective treatment and reduce unnecessary treatment, suffering, and the economic burden of cancer patients in the context of molecular diagnostics. The use of high‐throughput techniques combined with bioinformatics and systems biology has aided the interrogation of clinical samples and allowed the identification of molecular signatures that predict treatment responses. With the increase of drug response data in TCGA cohort, the sample expression and drug structural similarity network will become more extensive, which should make this approach more powerful. The two‐layer integrated network model can be used to predict the response not only to existing drugs but also to candidate drugs. At the present stage, our model is appropriate for predicting response of single drug, which is very important in precision medicine. To make our approach more powerful, we will extend the prediction model to multidrugs and concern on other types of ceRNA such as pseudogenes(Salmena *et al*., 2011) in our future study.

## 5 Conclusions

In this study, we proposed an integrative systems biology approach to predict individual drug responses based on DRCEs across multiple cancer types. We have indicated that DRCE dysregulation influenced drug response‐associated functions and pathways, suggesting DRCEs as potential therapeutic targets affecting drug responses. Our approach represents a powerful technique for understanding the underlying molecular mechanisms of drug responses and identifying novel therapeutic targets in cancer.

## Author contributions

Xia Li, YL, and SN conceived and designed the project. YZ conceived and designed the experiments and wrote the manuscript. Xin Li acquired and analyzed the data. DZ, HZ, and PW designed the algorithm. YG, MG, and MY performed the statistical analysis. YW and WS helped in interpreting the results.

## Supporting information


**Fig. S1.** Degree distribution of the DRCE networks across 10 types of cancer.
**Fig. S2.** Correlation‐density curves of LUSC DRCEs and non‐DRCEs.
**Fig. S3.** The percentage of lncRNA, miRNA and mRNA in DRCEs and non‐DRCEs whose correlation with simvastatin's drug activities more than 0.5, 0.4 and 0.3.
**Fig. S4.** Parameter optimization of the patient–drug two‐layer integrated network model.
**Fig. S5.** Scatter plots of observed and predicted DRS.
**Fig. S6.** Expression of six STAD DRCEs (hsa‐miR‐335_KLF8_LINC00641, hsa‐miR‐106b_APC_ENSG00000248175, hsa‐miR‐106b_APC_LINC01184, hsa‐miR‐106b_CCND2_ENSG00000248175, hsa‐miR‐331_ATRX_ENSG00000248175 and hsa‐miR‐331_ATRX_RP11‐473O4.3) in patients treated with 5‐FU.

**Table S1.** Molecular expression profiles for 13 TCGA major tumor types used in the study.

**Table S2.** The list of actual patient–drug pairs in each cancer.

**Table S3.** The DRCE ranking lists based on shared miRNA number in each cancer.

**Table S4.** The annotated GO terms of EGFR and SREBF1.

**Table S5.** Drug response‐associated pathways.

**Table S6.** The annotated GO terms of KLF8, ATRX, CCND2 and APC.
