# Supplementary material for: Inferences of individual drug responses across diverse cancer types using a novel competing endogenous RNA network
Source: Mol Oncol. 2018 Jul 14;12(9):1429–46. doi: 10.1002/1878-0261.12181 (PMC6120231; doi:10.1002/1878-0261.12181)
Supplement: Supplementary file 1 — Fig. S1. Degree distribution of the DRCE networks across 10 types of cancer. Fig. S2. Correlation‐density curves of LUSC DRCEs and non‐DRCEs. Fig. S3. The percentage of lncRNA, miRNA and mRNA in DRCEs and non‐DRCEs whose correlation with simvastatin's drug activities more than 0.5, 0.4 and 0.3. Fig. S4. Parameter optimization of the patient–drug two‐layer integrated network model. Fig. S5. Scatter plots of observed and predicted DRS. Fig. S6. Expression of six STAD DRCEs (hsa‐miR‐335_KLF8_LINC00641, hsa‐miR‐106b_APC_ENSG00000248175, hsa‐miR‐106b_APC_LINC01184, hsa‐miR‐106b_CCND2_ENSG00000248175, hsa‐miR‐331_ATRX_ENSG00000248175 and hsa‐miR‐331_ATRX_RP11‐473O4.3) in patients treated with 5‐FU. [file MOL2-12-1429-s001.pdf]

Supplemental files for “Inferences of individual drug responses across diverse cancer types using a novel competing endogenous RNA network”

Identification of ceRNAs across various cancer types

Human mature miRNA sequences were downloaded from the miRBase database (release 19) [1], and lncRNA sequences were obtained from the GENCODE database (v19) [2]. Previous studies indicated that lncRNAs could compete with mRNAs to bind miRNAs, which could then be detected using traditional miRNA target prediction algorithms [3-5]. The miRNA–lncRNA interactions were predicted using TargetScan (v.6.0) [6], PITA (March 2007 version) [7], miRanda (November 2010 version) [8] and RNAhybrid (v.2.1.1) [9] using the default parameters. To decrease the false positive rate of traditional prediction methods, miRNA–lncRNA interactions were filtered using AGO-CLIP-seq data [10], and then the experimentally validated miRNA–lncRNA interactions derived from DIANA-LncBase [10] and starBase v2.0 [11] were integrated. The miRNA–mRNA interactions were collected from TarBase (v6.0) [12] and mirTarBase (release 4.5) [13], which store manually curated collections of experimentally supported miRNA targets. For each cancer, the ceRNAs were identified by the following relationships. 1) miRNA–lncRNA and miRNA–mRNA interactions with a significant negative correlation of expression (Pearson correlation coefficient [PCC] < -0.3, $p < 0.05$) were retained [14, 15]. 2) The ceRNA triplet consisted of miRNA–lncRNA and miRNA–mRNA interactions sharing at least one miRNA [16]. 3) Expression of the lncRNA and mRNA in the ceRNA triplet were positively correlated (PCC > 0.3, $p < 0.05$) [17].

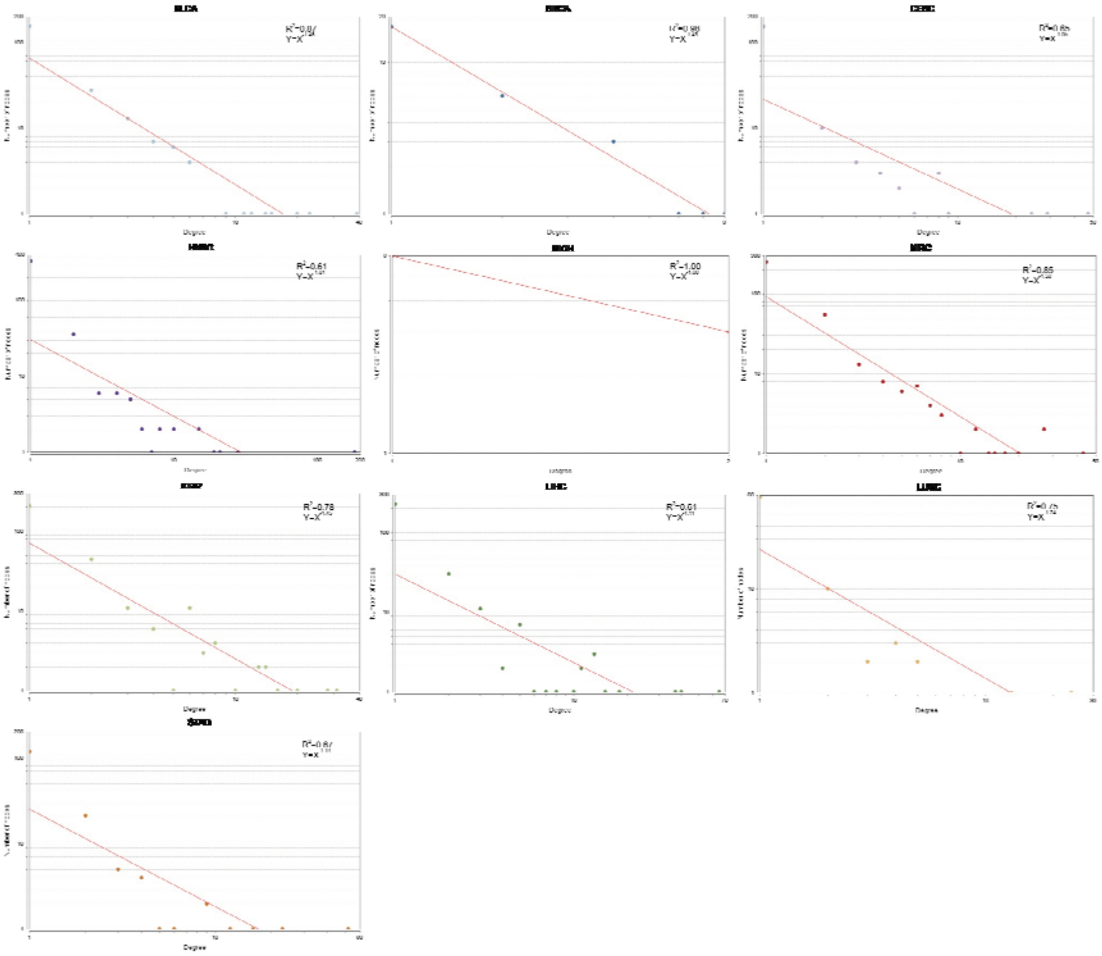

Figure S1. Degree distribution of the DRCE networks across 10 types of cancer. The x-axis is the ranked degree and the y-axis is the number of nodes at this rank. Most nodes are lowly connected and only a few are relatively highly connected. The examination of the degree distribution reveals a scale-free power-law distribution.

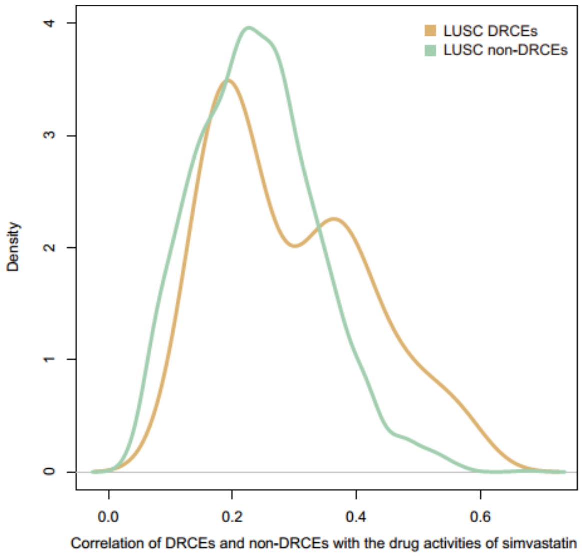

Figure S2. Correlation-density curves of LUSC DRCEs and non-DRCEs.

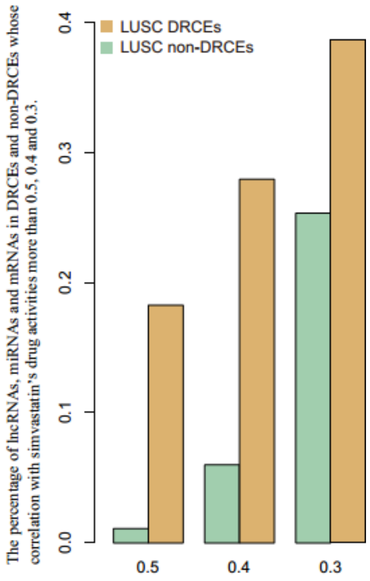

Figure S3. The percentage of lncRNAs, miRNAs and mRNAs in DRCEs and non-DRCEs whose correlation with simvastatin's drug activities more than 0.5, 0.4 and 0.3.

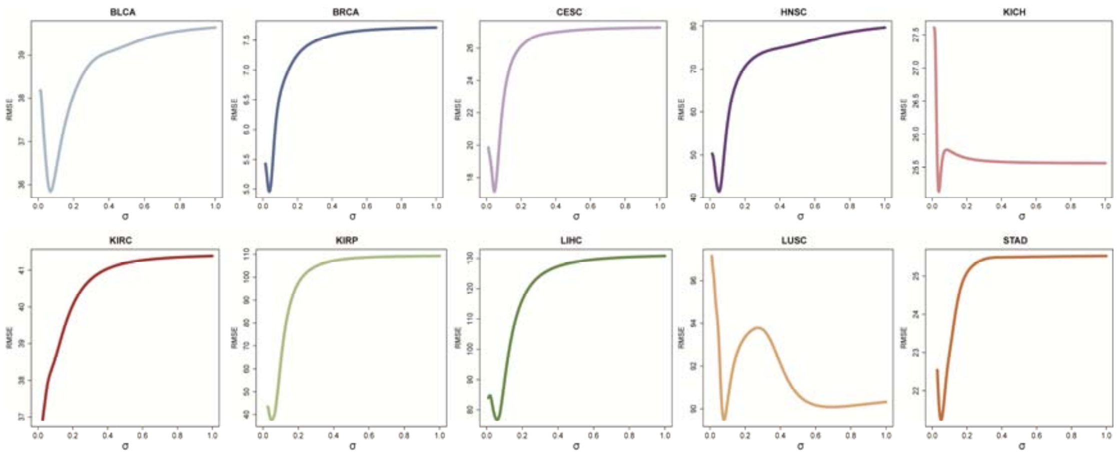

Figure S4. Parameter optimization of the patient-drug two-layer integrated network model. The RMSE range with a change in σ in 10 cancer types.

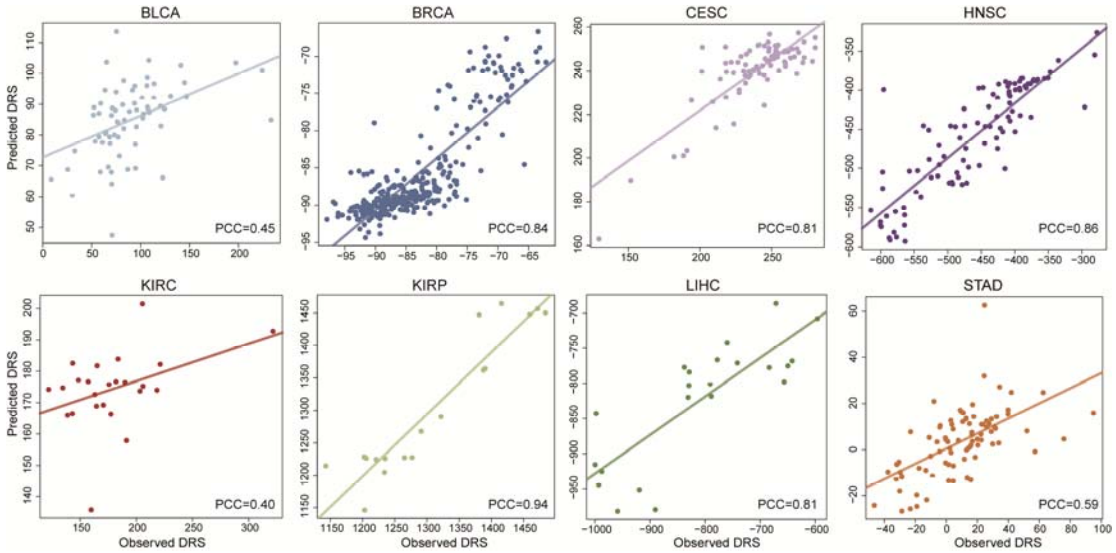

Figure S5. Scatter plots of observed and predicted DRS.

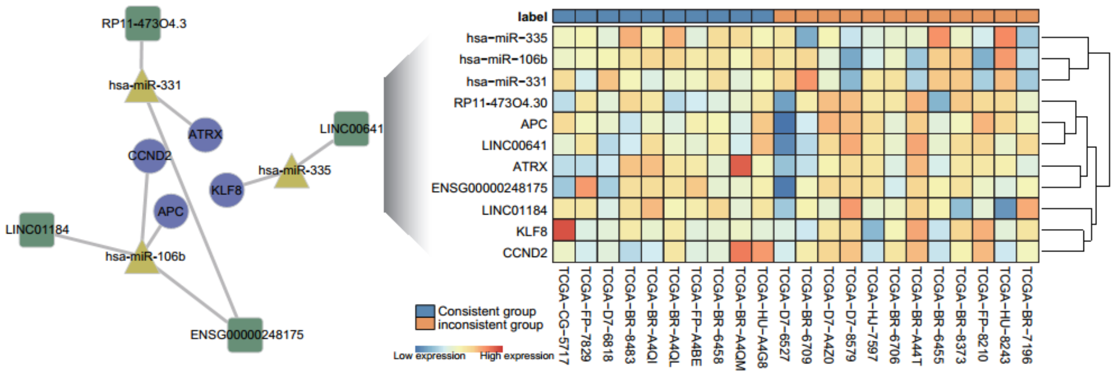

Figure S6. Expression of six STAD DRCEs (hsa-miR-335_KLF8_LINC00641, hsa-miR-106b_APC_ENSG00000248175, hsa-miR-106b_APC_LINC01184, hsa-miR-106b_CCND2_ENSG00000248175, hsa-miR-331_ATRX_ENSG00000248175 and hsa-miR-331_ATRX_RP11-473O4.3) in patients treated with 5-FU.

References

1. Kozomara, A. and S. Griffiths-Jones, *miRBase: integrating microRNA annotation and deep-sequencing data.* Nucleic Acids Res, 2011. **39**(Database issue): p. D152-7.
2. Derrien, T., et al., *The GENCODE v7 catalog of human long noncoding RNAs: analysis of their gene structure, evolution, and expression.* Genome Res, 2012. **22**(9): p. 1775-89.
3. Jeggari, A., D.S. Marks, and E. Larsson, *miRcode: a map of putative microRNA target sites in the long non-coding transcriptome.* Bioinformatics, 2012. **28**(15): p. 2062-3.
4. Liu, K., et al., *Linc2GO: a human LincRNA function annotation resource based on ceRNA hypothesis.* Bioinformatics, 2013. **29**(17): p. 2221-2.
5. Jalali, S., et al., *Systematic transcriptome wide analysis of lncRNA-miRNA interactions.* PLoS One, 2013. **8**(2): p. e53823.
6. Friedman, R.C., et al., *Most mammalian mRNAs are conserved targets of microRNAs.* Genome Res, 2009. **19**(1): p. 92-105.
7. Kertesz, M., et al., *The role of site accessibility in microRNA target recognition.* Nat Genet, 2007. **39**(10): p. 1278-84.
8. Betel, D., et al., *The microRNA.org resource: targets and expression.* Nucleic Acids Res, 2008. **36**(Database issue): p. D149-53.
9. Rehmsmeier, M., et al., *Fast and effective prediction of microRNA/target duplexes.* RNA, 2004. **10**(10): p. 1507-17.
10. Paraskevopoulou, M.D., et al., *DIANA-LncBase: experimentally verified and computationally predicted microRNA targets on long non-coding RNAs.* Nucleic Acids Res, 2013. **41**(Database issue): p. D239-45.
11. Li, J.H., et al., *starBase v2.0: decoding miRNA-ceRNA, miRNA-ncRNA and protein-RNA interaction networks from large-scale CLIP-Seq data.* Nucleic Acids Res, 2014. **42**(Database issue): p. D92-7.
12. Vergoulis, T., et al., *TarBase 6.0: capturing the exponential growth of miRNA targets with experimental support.* Nucleic Acids Res, 2012. **40**(Database issue): p. D222-9.
13. Hsu, S.D., et al., *miRTarBase update 2014: an information resource for experimentally validated miRNA-target interactions.* Nucleic Acids Res, 2014. **42**(Database issue): p. D78-85.
14. Tan, J.Y., et al., *Extensive microRNA-mediated crosstalk between lncRNAs and mRNAs in mouse embryonic stem cells.* Genome Res, 2015. **25**(5): p. 655-66.
15. Sumazin, P., et al., *An extensive microRNA-mediated network of RNA-RNA interactions regulates established oncogenic pathways in glioblastoma.* Cell, 2011. **147**(2): p. 370-81.
16. Ala, U., et al., *Integrated transcriptional and competitive endogenous RNA networks are cross-regulated in permissive molecular environments.* Proc Natl Acad Sci U S A, 2013. **110**(18): p. 7154-9.
17. Karreth, F.A., et al., *In vivo identification of tumor- suppressive PTEN ceRNAs in an oncogenic BRAF-induced mouse model of melanoma.* Cell, 2011. **147**(2): p. 382-95.
